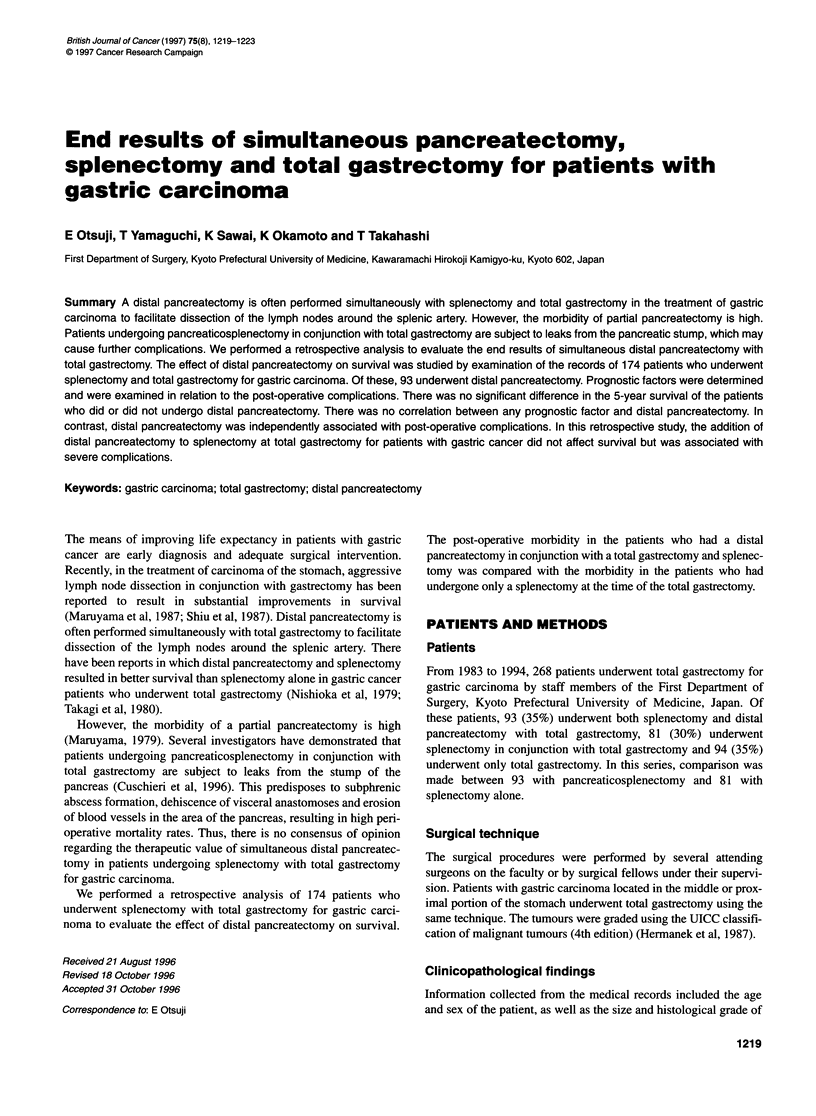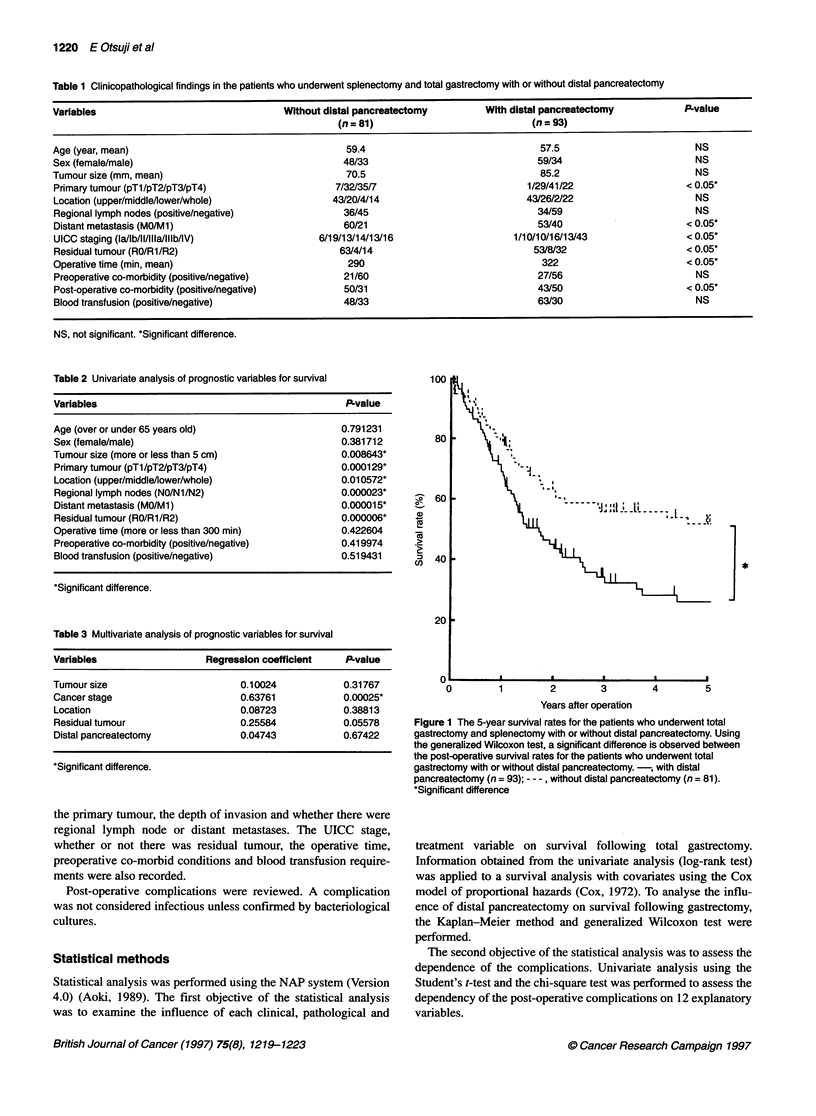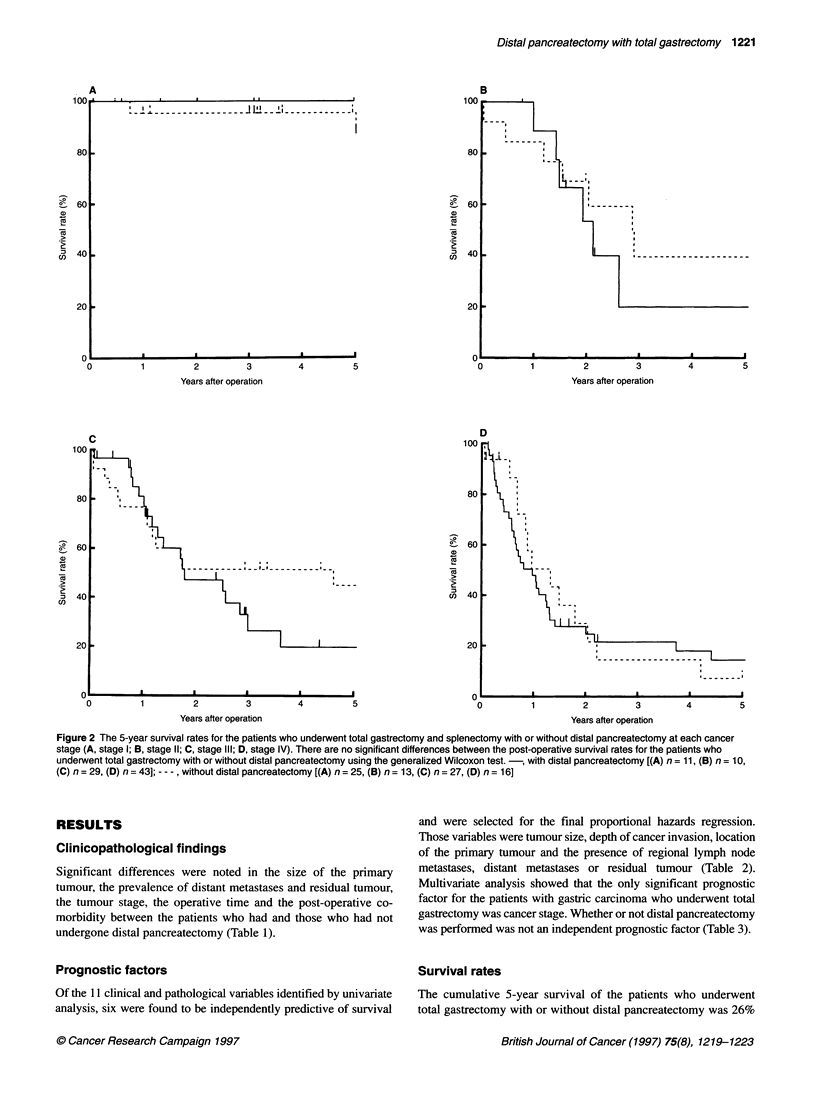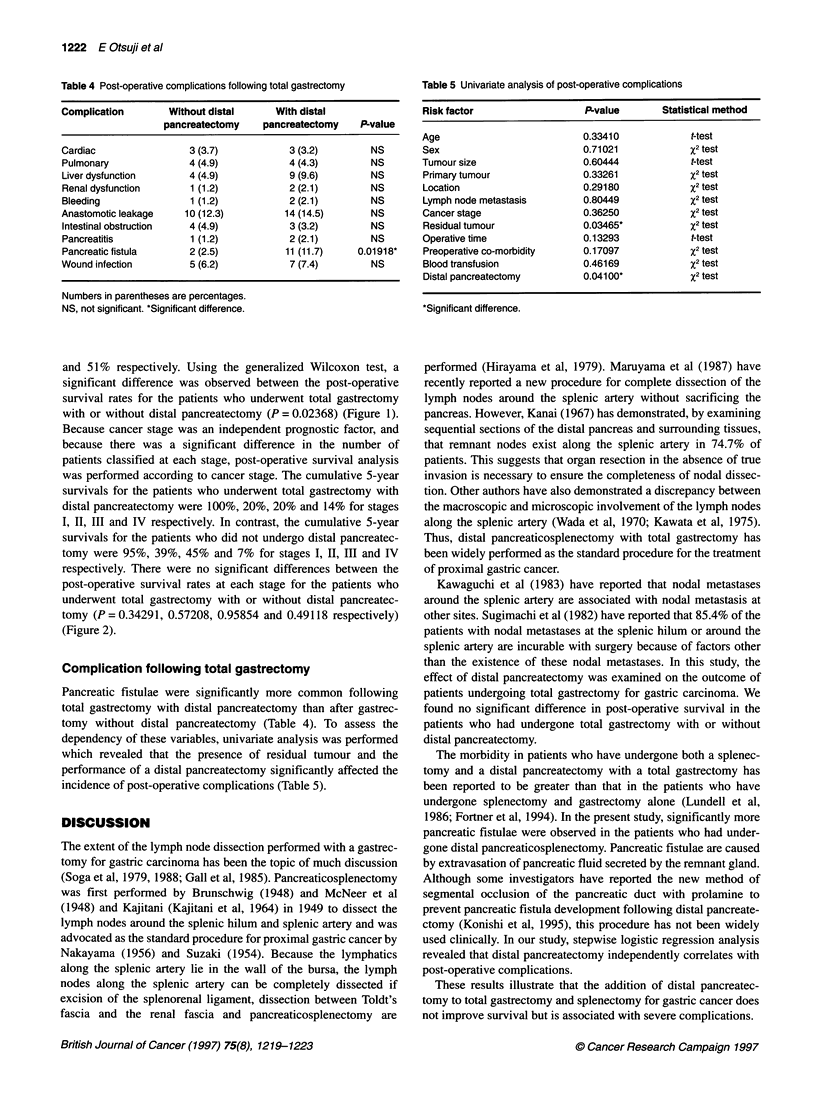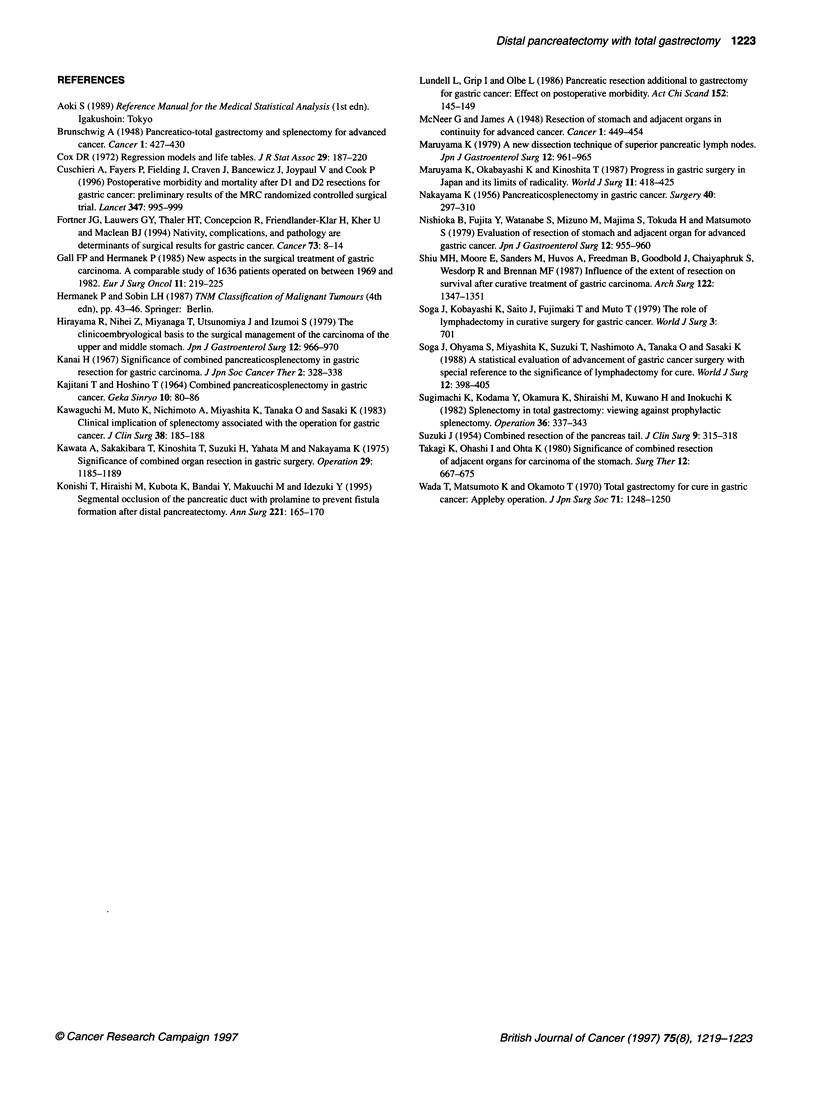# End results of simultaneous pancreatectomy, splenectomy and total gastrectomy for patients with gastric carcinoma.

**DOI:** 10.1038/bjc.1997.209

**Published:** 1997

**Authors:** E. Otsuji, T. Yamaguchi, K. Sawai, K. Okamoto, T. Takahashi

**Affiliations:** First Department of Surgery, Kyoto Prefectural University of Medicine, Kawaramachi Hirokoji Kamigyo-ku, Japan.

## Abstract

A distal pancreatectomy is often performed simultaneously with splenectomy and total gastrectomy in the treatment of gastric carcinoma to facilitate dissection of the lymph nodes around the splenic artery. However, the morbidity of partial pancreatectomy is high. Patients undergoing pancreaticosplenectomy in conjunction with total gastrectomy are subject to leaks from the pancreatic stump, which may cause further complications. We performed a retrospective analysis to evaluate the end results of simultaneous distal pancreatectomy with total gastrectomy. The effect of distal pancreatectomy on survival was studied by examination of the records of 174 patients who underwent splenectomy and total gastrectomy for gastric carcinoma. Of these, 93 underwent distal pancreatectomy. Prognostic factors were determined and were examined in relation to the post-operative complications. There was no significant difference in the 5-year survival of the patients who did or did not undergo distal pancreatectomy. There was no correlation between any prognostic factor and distal pancreatectomy. In contrast, distal pancreatectomy was independently associated with post-operative complications. In this retrospective study, the addition of distal pancreatectomy to splenectomy at total gastrectomy for patients with gastric cancer did not affect survival but was associated with severe complications.


British Journal of Cancer (1997) 75(8), 1219–1223
© 1997 Cancer Research Campaign

### End results of simultaneous pancreatectomy, splenectomy and total gastrectomy for patients with gastric carcinoma

**E Otsuji, T Yamaguchi, K Sawai, K Okamoto and T Takahashi**

First Department of Surgery, Kyoto Prefectural University of Medicine, Kawaramachi Hirokoji Kamigyo-ku, Kyoto 602, Japan

**Summary** A distal pancreatectomy is often performed simultaneously with splenectomy and total gastrectomy in the treatment of gastric carcinoma to facilitate dissection of the lymph nodes around the splenic artery. However, the morbidity of partial pancreatectomy is high. Patients undergoing pancreaticosplenectomy in conjunction with total gastrectomy are subject to leaks from the pancreatic stump, which may cause further complications. We performed a retrospective analysis to evaluate the end results of simultaneous distal pancreatectomy with total gastrectomy. The effect of distal pancreatectomy on survival was studied by examination of the records of 174 patients who underwent splenectomy and total gastrectomy for gastric carcinoma. Of these, 93 underwent distal pancreatectomy. Prognostic factors were determined and were examined in relation to the post-operative complications. There was no significant difference in the 5-year survival of the patients who did or did not undergo distal pancreatectomy. There was no correlation between any prognostic factor and distal pancreatectomy. In contrast, distal pancreatectomy was independently associated with post-operative complications. In this retrospective study, the addition of distal pancreatectomy to splenectomy at total gastrectomy for patients with gastric cancer did not affect survival but was associated with severe complications.

**Keywords:** gastric carcinoma; total gastrectomy; distal pancreatectomy

The means of improving life expectancy in patients with gastric cancer are early diagnosis and adequate surgical intervention. Recently, in the treatment of carcinoma of the stomach, aggressive lymph node dissection in conjunction with gastrectomy has been reported to result in substantial improvements in survival (Maruyama et al, 1987; Shiu et al, 1987). Distal pancreatectomy is often performed simultaneously with total gastrectomy to facilitate dissection of the lymph nodes around the splenic artery. There have been reports in which distal pancreatectomy and splenectomy resulted in better survival than splenectomy alone in gastric cancer patients who underwent total gastrectomy (Nishioka et al, 1979; Takagi et al, 1980).

However, the morbidity of a partial pancreatectomy is high (Maruyama, 1979). Several investigators have demonstrated that patients undergoing pancreaticosplenectomy in conjunction with total gastrectomy are subject to leaks from the stump of the pancreas (Cuschieri et al, 1996). This predisposes to subphrenic abscess formation, dehiscence of visceral anastomoses and erosion of blood vessels in the area of the pancreas, resulting in high peri-operative mortality rates. Thus, there is no consensus of opinion regarding the therapeutic value of simultaneous distal pancreatectomy in patients undergoing splenectomy with total gastrectomy for gastric carcinoma.

We performed a retrospective analysis of 174 patients who underwent splenectomy with total gastrectomy for gastric carcinoma to evaluate the effect of distal pancreatectomy on survival. The post-operative morbidity in the patients who had a distal pancreatectomy in conjunction with a total gastrectomy and splenectomy was compared with the morbidity in the patients who had undergone only a splenectomy at the time of the total gastrectomy.

*Received 21 August 1996*
*Revised 18 October 1996*
*Accepted 31 October 1996*

*Correspondence to:* E Otsuji

### PATIENTS AND METHODS

### Patients

From 1983 to 1994, 268 patients underwent total gastrectomy for gastric carcinoma by staff members of the First Department of Surgery, Kyoto Prefectural University of Medicine, Japan. Of these patients, 93 (35%) underwent both splenectomy and distal pancreatectomy with total gastrectomy, 81 (30%) underwent splenectomy in conjunction with total gastrectomy and 94 (35%) underwent only total gastrectomy. In this series, comparison was made between 93 with pancreaticosplenectomy and 81 with splenectomy alone.

### Surgical technique

The surgical procedures were performed by several attending surgeons on the faculty or by surgical fellows under their supervision. Patients with gastric carcinoma located in the middle or proximal portion of the stomach underwent total gastrectomy using the same technique. The tumours were graded using the UICC classification of malignant tumours (4th edition) (Hermanek et al, 1987).

### Clinicopathological findings

Information collected from the medical records included the age and sex of the patient, as well as the size and histological grade of

Table 1 Clinicopathological findings in the patients who underwent splenectomy and total gastrectomy with or without distal pancreatectomy

| Variables | Without distal pancreatectomy ($n$ = 81) | With distal pancreatectomy ($n$ = 93) | $P$-value |
|---|---|---|---|
| Age (year, mean) | 59.4 | 57.5 | NS |
| Sex (female/male) | 48/33 | 59/34 | NS |
| Tumour size (mm, mean) | 70.5 | 85.2 | NS |
| Primary tumour (pT1/pT2/pT3/pT4) | 7/32/35/7 | 1/29/41/22 | < 0.05* |
| Location (upper/middle/lower/whole) | 43/20/4/14 | 43/26/2/22 | NS |
| Regional lymph nodes (positive/negative) | 36/45 | 34/59 | NS |
| Distant metastasis (M0/M1) | 60/21 | 53/40 | < 0.05* |
| UICC staging (Ia/Ib/II/IIIa/IIIb/IV) | 6/19/13/14/13/16 | 1/10/10/16/13/43 | < 0.05* |
| Residual tumour (R0/R1/R2) | 63/4/14 | 53/8/32 | < 0.05* |
| Operative time (min, mean) | 290 | 322 | < 0.05* |
| Preoperative co-morbidity (positive/negative) | 21/60 | 27/56 | NS |
| Post-operative co-morbidity (positive/negative) | 50/31 | 43/50 | < 0.05* |
| Blood transfusion (positive/negative) | 48/33 | 63/30 | NS |

NS, not significant. *Significant difference.

Table 2 Univariate analysis of prognostic variables for survival

| Variables | $P$-value |
|---|---|
| Age (over or under 65 years old) | 0.791231 |
| Sex (female/male) | 0.381712 |
| Tumour size (more or less than 5 cm) | 0.008643* |
| Primary tumour (pT1/pT2/pT3/pT4) | 0.000129* |
| Location (upper/middle/lower/whole) | 0.010572* |
| Regional lymph nodes (N0/N1/N2) | 0.000023* |
| Distant metastasis (M0/M1) | 0.000015* |
| Residual tumour (R0/R1/R2) | 0.000006* |
| Operative time (more or less than 300 min) | 0.422604 |
| Preoperative co-morbidity (positive/negative) | 0.419974 |
| Blood transfusion (positive/negative) | 0.519431 |

*Significant difference.

Table 3 Multivariate analysis of prognostic variables for survival

| Variables | Regression coefficient | $P$-value |
|---|---|---|
| Tumour size | 0.10024 | 0.31767 |
| Cancer stage | 0.63761 | 0.00025* |
| Location | 0.08723 | 0.38813 |
| Residual tumour | 0.25584 | 0.05578 |
| Distal pancreatectomy | 0.04743 | 0.67422 |

*Significant difference.

Figure 1 The 5-year survival rates for the patients who underwent total gastrectomy and splenectomy with or without distal pancreatectomy. Using the generalized Wilcoxon test, a significant difference is observed between the post-operative survival rates for the patients who underwent total gastrectomy with or without distal pancreatectomy. —, with distal pancreatectomy ($n$ = 93); - - - , without distal pancreatectomy ($n$ = 81). *Significant difference the primary tumour, the depth of invasion and whether there were regional lymph node or distant metastases. The UICC stage, whether or not there was residual tumour, the operative time, preoperative co-morbid conditions and blood transfusion requirements were also recorded.

Post-operative complications were reviewed. A complication was not considered infectious unless confirmed by bacteriological cultures.

### Statistical methods

Statistical analysis was performed using the NAP system (Version 4.0) (Aoki, 1989). The first objective of the statistical analysis was to examine the influence of each clinical, pathological and treatment variable on survival following total gastrectomy. Information obtained from the univariate analysis (log-rank test) was applied to a survival analysis with covariates using the Cox model of proportional hazards (Cox, 1972). To analyse the influence of distal pancreatectomy on survival following gastrectomy, the Kaplan–Meier method and generalized Wilcoxon test were performed.

The second objective of the statistical analysis was to assess the dependence of the complications. Univariate analysis using the Student's $t$-test and the chi-square test was performed to assess the dependency of the post-operative complications on 12 explanatory variables.

© Cancer Research Campaign 1997

**Figure 2** The 5-year survival rates for the patients who underwent total gastrectomy and splenectomy with or without distal pancreatectomy at each cancer stage (**A**, stage I; **B**, stage II; **C**, stage III; **D**, stage IV). There are no significant differences between the post-operative survival rates for the patients who underwent total gastrectomy with or without distal pancreatectomy using the generalized Wilcoxon test. —, with distal pancreatectomy [(**A**) *n* = 11, (**B**) *n* = 10, (**C**) *n* = 29, (**D**) *n* = 43]; - - - , without distal pancreatectomy [(**A**) *n* = 25, (**B**) *n* = 13, (**C**) *n* = 27, (**D**) *n* = 16]

### RESULTS

### Clinicopathological findings

Significant differences were noted in the size of the primary tumour, the prevalence of distant metastases and residual tumour, the tumour stage, the operative time and the post-operative co-morbidity between the patients who had and those who had not undergone distal pancreatectomy (Table 1).

### Prognostic factors

Of the 11 clinical and pathological variables identified by univariate analysis, six were found to be independently predictive of survival and were selected for the final proportional hazards regression. Those variables were tumour size, depth of cancer invasion, location of the primary tumour and the presence of regional lymph node metastases, distant metastases or residual tumour (Table 2). Multivariate analysis showed that the only significant prognostic factor for the patients with gastric carcinoma who underwent total gastrectomy was cancer stage. Whether or not distal pancreatectomy was performed was not an independent prognostic factor (Table 3).

### Survival rates

The cumulative 5-year survival of the patients who underwent total gastrectomy with or without distal pancreatectomy was 26%

**Table 4** Post-operative complications following total gastrectomy

| Complication | Without distal pancreatectomy | With distal pancreatectomy | P-value |
|---|---|---|---|
| Cardiac | 3 (3.7) | 3 (3.2) | NS |
| Pulmonary | 4 (4.9) | 4 (4.3) | NS |
| Liver dysfunction | 4 (4.9) | 9 (9.6) | NS |
| Renal dysfunction | 1 (1.2) | 2 (2.1) | NS |
| Bleeding | 1 (1.2) | 2 (2.1) | NS |
| Anastomotic leakage | 10 (12.3) | 14 (14.5) | NS |
| Intestinal obstruction | 4 (4.9) | 3 (3.2) | NS |
| Pancreatitis | 1 (1.2) | 2 (2.1) | NS |
| Pancreatic fistula | 2 (2.5) | 11 (11.7) | 0.01918* |
| Wound infection | 5 (6.2) | 7 (7.4) | NS |

Numbers in parentheses are percentages.
NS, not significant. *Significant difference.

**Table 5** Univariate analysis of post-operative complications

| Risk factor | P-value | Statistical method |
|---|---|---|
| Age | 0.33410 | t-test |
| Sex | 0.71021 | $\chi^2$ test |
| Tumour size | 0.60444 | t-test |
| Primary tumour | 0.33261 | $\chi^2$ test |
| Location | 0.29180 | $\chi^2$ test |
| Lymph node metastasis | 0.80449 | $\chi^2$ test |
| Cancer stage | 0.36250 | $\chi^2$ test |
| Residual tumour | 0.03465* | $\chi^2$ test |
| Operative time | 0.13293 | t-test |
| Preoperative co-morbidity | 0.17097 | $\chi^2$ test |
| Blood transfusion | 0.46169 | $\chi^2$ test |
| Distal pancreatectomy | 0.04100* | $\chi^2$ test |

*Significant difference.

and 51% respectively. Using the generalized Wilcoxon test, a significant difference was observed between the post-operative survival rates for the patients who underwent total gastrectomy with or without distal pancreatectomy ($P = 0.02368$) (Figure 1). Because cancer stage was an independent prognostic factor, and because there was a significant difference in the number of patients classified at each stage, post-operative survival analysis was performed according to cancer stage. The cumulative 5-year survivals for the patients who underwent total gastrectomy with distal pancreatectomy were 100%, 20%, 20% and 14% for stages I, II, III and IV respectively. In contrast, the cumulative 5-year survivals for the patients who did not undergo distal pancreatectomy were 95%, 39%, 45% and 7% for stages I, II, III and IV respectively. There were no significant differences between the post-operative survival rates at each stage for the patients who underwent total gastrectomy with or without distal pancreatectomy ($P = 0.34291$, 0.57208, 0.95854 and 0.49118 respectively) (Figure 2).

### Complication following total gastrectomy

Pancreatic fistulae were significantly more common following total gastrectomy with distal pancreatectomy than after gastrectomy without distal pancreatectomy (Table 4). To assess the dependency of these variables, univariate analysis was performed which revealed that the presence of residual tumour and the performance of a distal pancreatectomy significantly affected the incidence of post-operative complications (Table 5).

### DISCUSSION

The extent of the lymph node dissection performed with a gastrectomy for gastric carcinoma has been the topic of much discussion (Soga et al, 1979, 1988; Gall et al, 1985). Pancreaticosplenectomy was first performed by Brunschwig (1948) and McNeer et al (1948) and Kajitani (Kajitani et al, 1964) in 1949 to dissect the lymph nodes around the splenic hilum and splenic artery and was advocated as the standard procedure for proximal gastric cancer by Nakayama (1956) and Suzaki (1954). Because the lymphatics along the splenic artery lie in the wall of the bursa, the lymph nodes along the splenic artery can be completely dissected if excision of the splenorenal ligament, dissection between Toldt's fascia and the renal fascia and pancreaticosplenectomy are performed (Hirayama et al, 1979). Maruyama et al (1987) have recently reported a new procedure for complete dissection of the lymph nodes around the splenic artery without sacrificing the pancreas. However, Kanai (1967) has demonstrated, by examining sequential sections of the distal pancreas and surrounding tissues, that remnant nodes exist along the splenic artery in 74.7% of patients. This suggests that organ resection in the absence of true invasion is necessary to ensure the completeness of nodal dissection. Other authors have also demonstrated a discrepancy between the macroscopic and microscopic involvement of the lymph nodes along the splenic artery (Wada et al, 1970; Kawata et al, 1975). Thus, distal pancreaticosplenectomy with total gastrectomy has been widely performed as the standard procedure for the treatment of proximal gastric cancer.

Kawaguchi et al (1983) have reported that nodal metastases around the splenic artery are associated with nodal metastasis at other sites. Sugimachi et al (1982) have reported that 85.4% of the patients with nodal metastases at the splenic hilum or around the splenic artery are incurable with surgery because of factors other than the existence of these nodal metastases. In this study, the effect of distal pancreatectomy was examined on the outcome of patients undergoing total gastrectomy for gastric carcinoma. We found no significant difference in post-operative survival in the patients who had undergone total gastrectomy with or without distal pancreatectomy.

The morbidity in patients who have undergone both a splenectomy and a distal pancreatectomy with a total gastrectomy has been reported to be greater than that in the patients who have undergone splenectomy and gastrectomy alone (Lundell et al, 1986; Fortner et al, 1994). In the present study, significantly more pancreatic fistulae were observed in the patients who had undergone distal pancreaticosplenectomy. Pancreatic fistulae are caused by extravasation of pancreatic fluid secreted by the remnant gland. Although some investigators have reported the new method of segmental occlusion of the pancreatic duct with prolamine to prevent pancreatic fistula development following distal pancreatectomy (Konishi et al, 1995), this procedure has not been widely used clinically. In our study, stepwise logistic regression analysis revealed that distal pancreatectomy independently correlates with post-operative complications.

These results illustrate that the addition of distal pancreatectomy to total gastrectomy and splenectomy for gastric cancer does not improve survival but is associated with severe complications.

© Cancer Research Campaign 1997

### REFERENCES

Aoki S (1989) *Reference Manual for the Medical Statistical Analysis* (1st edn). Igakushoin: Tokyo

Brunschwig A (1948) Pancreatico-total gastrectomy and splenectomy for advanced cancer. *Cancer* **1**: 427–430

Cox DR (1972) Regression models and life tables. *J R Stat Assoc* **29**: 187–220

Cuschieri A, Fayers P, Fielding J, Craven J, Bancewicz J, Joypaul V and Cook P (1996) Postoperative morbidity and mortality after D1 and D2 resections for gastric cancer: preliminary results of the MRC randomized controlled surgical trial. *Lancet* **347**: 995–999

Fortner JG, Lauwers GY, Thaler HT, Concepcion R, Friendlander-Klar H, Kher U and Maclean BJ (1994) Nativity, complications, and pathology are determinants of surgical results for gastric cancer. *Cancer* **73**: 8–14

Gall FP and Hermanek P (1985) New aspects in the surgical treatment of gastric carcinoma. A comparable study of 1636 patients operated on between 1969 and 1982. *Eur J Surg Oncol* **11**: 219–225

Hermanek P and Sobin LH (1987) *TNM Classification of Malignant Tumours* (4th edn), pp. 43–46. Springer: Berlin.

Hirayama R, Nihei Z, Miyanaga T, Utsunomiya J and Izumoi S (1979) The clinicoembryological basis to the surgical management of the carcinoma of the upper and middle stomach. *Jpn J Gastroenterol Surg* **12**: 966–970

Kanai H (1967) Significance of combined pancreaticosplenectomy in gastric resection for gastric carcinoma. *J Jpn Soc Cancer Ther* **2**: 328–338

Kajitani T and Hoshino T (1964) Combined pancreaticosplenectomy in gastric cancer. *Geka Sinryo* **10**: 80–86

Kawaguchi M, Muto K, Nichimoto A, Miyashita K, Tanaka O and Sasaki K (1983) Clinical implication of splenectomy associated with the operation for gastric cancer. *J Clin Surg* **38**: 185–188

Kawata A, Sakakibara T, Kinoshita T, Suzuki H, Yahata M and Nakayama K (1975) Significance of combined organ resection in gastric surgery. *Operation* **29**: 1185–1189

Konishi T, Hiraishi M, Kubota K, Bandai Y, Makuuchi M and Idezuki Y (1995) Segmental occlusion of the pancreatic duct with prolamine to prevent fistula formation after distal pancreatectomy. *Ann Surg* **221**: 165–170

Lundell L, Grip I and Olbe L (1986) Pancreatic resection additional to gastrectomy for gastric cancer: Effect on postoperative morbidity. *Act Chi Scand* **152**: 145–149

McNeer G and James A (1948) Resection of stomach and adjacent organs in continuity for advanced cancer. *Cancer* **1**: 449–454

Maruyama K (1979) A new dissection technique of superior pancreatic lymph nodes. *Jpn J Gastroenterol Surg* **12**: 961–965

Maruyama K, Okabayashi K and Kinoshita T (1987) Progress in gastric surgery in Japan and its limits of radicality. *World J Surg* **11**: 418–425

Nakayama K (1956) Pancreaticosplenectomy in gastric cancer. *Surgery* **40**: 297–310

Nishioka B, Fujita Y, Watanabe S, Mizuno M, Majima S, Tokuda H and Matsumoto S (1979) Evaluation of resection of stomach and adjacent organ for advanced gastric cancer. *Jpn J Gastroenterol Surg* **12**: 955–960

Shiu MH, Moore E, Sanders M, Huvos A, Freedman B, Goodbold J, Chaiyaphruk S, Wesdorp R and Brennan MF (1987) Influence of the extent of resection on survival after curative treatment of gastric carcinoma. *Arch Surg* **122**: 1347–1351

Soga J, Kobayashi K, Saito J, Fujimaki T and Muto T (1979) The role of lymphadectomy in curative surgery for gastric cancer. *World J Surg* **3**: 701

Soga J, Ohyama S, Miyashita K, Suzuki T, Nashimoto A, Tanaka O and Sasaki K (1988) A statistical evaluation of advancement of gastric cancer surgery with special reference to the significance of lymphadectomy for cure. *World J Surg* **12**: 398–405

Sugimachi K, Kodama Y, Okamura K, Shiraishi M, Kuwano H and Inokuchi K (1982) Splenectomy in total gastrectomy: viewing against prophylactic splenectomy. *Operation* **36**: 337–343

Suzuki J (1954) Combined resection of the pancreas tail. *J Clin Surg* **9**: 315–318

Takagi K, Ohashi I and Ohta K (1980) Significance of combined resection of adjacent organs for carcinoma of the stomach. *Surg Ther* **12**: 667–675

Wada T, Matsumoto K and Okamoto T (1970) Total gastrectomy for cure in gastric cancer: Appleby operation. *J Jpn Surg Soc* **71**: 1248–1250

© Cancer Research Campaign 1997